# More data needed on neonatal microbiome seeding

**DOI:** 10.1186/s40168-022-01282-3

**Published:** 2022-06-10

**Authors:** W. Florian Fricke, Jacques Ravel

**Affiliations:** 1 Department of Microbiome Research and Applied Bioinformatics, University of Hohenheim, Stuttgart, Germany; 2 Institute for Genome Sciences, University of Maryland School of Medicine, Baltimore, MD USA

A convincing body of epidemiological research has linked birth by Cesarean section (C-section) with an increased risk of infectious and chronic diseases such as autoimmune diseases, asthma, and obesity, and even some cancers [1–4]. Compared to vaginal delivery, birth by C-section is often associated with alterations in neonatal intestinal microbiota diversity, composition, and developmental trajectories [5], and thus one hypothesis put forward is that C-section disrupts the vertical transmission of beneficial microbes from the mother to the infant during a critical developmental window [6–8]. However, major gaps remain in our understanding of the processes involved in early microbiota initialization and maturation, and a direct link between impaired maternal microbiota transfer at birth and acute or chronic illnesses in infants born by C-section has not been established and mechanistically explained.

Microbiota restoration for infants born by C-section has been proposed, tested for safety in small proof-of-concept clinical trials, and evaluated based on the efficacy to compensate for a lack of maternal microbiota transfer during birth. Proposed therapies include “vaginal seeding”, which is based on the inoculation of newborns with a microbiome sample collected from the mother’s vagina before birth [9], and maternal fecal microbiota transplantation, i.e. the supplementation of breast milk with a fecal bacterial suspension from the mother [10]. Both approaches are conceptually appealing, as they could reestablish vertical routes of microbial transmission interrupted by C-section and induce substantial and long-term effects on intestinal microbiome development, organization and function in the infants. Not surprisingly, they have received widespread attention in the scientific and lay press, leading parents preparing for C-section to inquire about vaginal seeding options at the hospital or at home [11]. Yet, most medical societies do not recommend the procedures due to the lack of strong data supporting their efficacy [12, 13], and the topic remains controversial.

To contribute to the ongoing debate and provide a knowledge base for a broader scientific, clinical and public discussion of microbiome restoration therapies in infants born by C-section, *Microbiome* has asked several experts in the field to present their perspectives on the biological mechanisms, clinical relevance and potential for therapeutic restoration of the neonatal microbiome [14]. Here, we identify key questions and knowledge gaps that should be addressed in order to better define microbiome deficits, their link to health outcomes and appropriate clinical intervention strategies in the neonatal patient population.

## How important is the birth type for microbiome establishment and maturation in the context of other contributing factors?

Exposure of infants to maternal microbiomes during vaginal birth represents only one of several perinatal and early-life contributors to neonatal microbiota initialization and development. While prenatal microbiota colonization *in utero* remains controversial and likely non-significant under non-pathological conditions (see *Microbiome*’s special issue on this topic: [15]), there may be other, indirect maternal influences during pregnancy on the neonatal microbiota, as probiotic supplementation [16] or enteric infection [17] in pregnant mice have been shown to affect offspring immunity. After birth, cross-fostering [18] and cohousing [19] in mice, as well as postnatal acquisition of maternal and paternal microbial strains during the first weeks, months and years after birth [7], and breastfeeding [5, 20] among others, have been shown to affect the trajectory of microbiota maturation and immune system development, and may, at least to some extent, compensate for a lack of maternal microbiota transfer during C-section.

## Does C-section temporarily disrupt or permanently impair microbiota development, with immediate or lasting consequences?

Most published studies have reported major compositional microbiota differences between vaginally delivered and C-section infants during the first weeks and months of life that disappear between 2 and 5 years of age, as the infant microbiota matures towards an adult state [5, 7, 21, 22], although some studies have reported detectable microbiota signatures in older (5-7 year-old) children born by C-section [21, 23]. An ecological vacancy in the neonatal microbiota after birth, due to reduced maternal microbiota transfer, could result in immediate metabolic or immune consequences that may increase the risk for acute pathologies, such as increased colonization with opportunistic pathogens [6] or may affect microbial or human developmental processes with health consequences later in life. Intestinal microbiota maturation in infants born vaginally or by C-section appears to follow distinct, but converging developmental trajectories [7]. Yet experimental data from mice suggest lasting health consequences from even transient compositional microbiota alterations, as antibiotic perturbation during a critical developmental window induced lasting metabolic deficits [24] and transfer of a wild mice-derived microbiota via fostering or cohousing during early pre-weaning life, protected mice from obesity-associated phenotypes [25]. In addition, compositional similarities at the genus or species level, *i.e.,* the taxonomic resolution typically achieved with 16S rRNA gene amplicon or metagenome sequencing, may obscure microbiota alterations at the strain level. Instead of receiving optimal microbial strains from their mothers, infants born by C-section could obtain alternative, suboptimal strains from other, alternative sources, including nutrition [20]. This could result in acute impaired metabolic capabilities, as strain-specific genotype variations with consequences for oligosaccharides utilization for example, have been reported for maternally transferred dominant and secondary *Bifidobacterium* strains [26]. It should also be noted that members of the genera *Bacteroides* and *Parabacteroides*, which are frequently transferred from mothers during vaginal birth [6, 7, 26, 27], often persist over time in the gut of healthy adults [28] and persistence patterns of these taxa are strongly linked to family and geography [29], suggesting a potential for co-evolution of specifically adapted bacterial strains from these genera with the human host, as seen for other bacteria [30]. These transmission and co-evolution processes may be disrupted by C-section birth, potentially resulting in lifelong microbiome alterations.

## How does C-section quantitatively affect neonatal microbiome dynamics?

There have only been a few attempts to quantify the neonatal microbiome in terms of absolute intestinal microbial abundances (microbial load), but with unexpected and interesting results. Quantitative fecal microbiota profiling of preterm infants identified bacterial and fungal blooms and extinctions, which would not have been detectable with standard, qualitative microbiota analysis methods such as 16S rRNA gene amplicon sequencing [31]. For example, decreased relative abundances of staphylococci in preterm infants would be wrongfully interpreted as suggesting reduced colonization, when in fact constant absolute abundances of this genus indicated stable colonization in the context of increased overall microbial loads [31]. The impaired transfer of the maternal microbiota during C-section, particularly of intestinal bacteria, has been associated with an increased fecal relative abundance of opportunistic pathogens [6]. However, exposure to the same bacterial species from skin, hospital and other environmental sources may also be expected for other newborns, and vaginally delivered infants do harbor such opportunistic pathogens in their feces, albeit at reduced relative abundances [7]. Thus, increased relative abundances of opportunistic bacteria in infants born by C-section do not necessarily indicate their bloom, but could merely reflect a lack of other intestinal microbes. This distinction may have clinical implications, as a quantitative over-abundance of opportunistic pathogens may be prevented by reducing exposure to these bacteria or depleting them with antibiotics, whereas vaginal seeding or fecal microbiota transfer (FMT)-like treatment therapies would be more suitable to compensate for the absence of commensal bacteria.

## Is the maternal vaginal or intestinal microbiota the most important source for neonatal microbiota inoculation?

Vaginal bacteria and maternal vaginal strains constitute only a small and transient fraction of the neonatal intestinal microbiota after birth, whereas intestinal microbes and maternal intestinal strains contribute a larger and more persistent portion [7, 27, 32]. Success in restoring taxonomic fecal microbiota compositions in infants born by C-section, which more closely resemble those of vaginally born infants, including restoring disrupted transmission of intestinal *Bacteroides* strains, has been reported both for vaginal seeding [9, 33] and maternal fecal microbiota transplantation [10]. It is conceivable that both vaginal and intestinal bacteria exert distinct, non-overlapping influences on the developing neonatal microbiota. However, oral administration of the maternal vaginal microbiota to C-section infants had no discernible effect on microbiota composition and rarely resulted in the engraftment of maternal strains in treated infants [34]. Interestingly, the identification of intestinal bacteria in vaginal fluids at the time of birth [33] suggests a temporal permeability of vaginal and intestinal microbiome boundaries, which may be evolutionarily intended to facilitate vertical microbiota transfer. It is also noteworthy that *Bacteroides* strains were among the most frequently engrafted members of the donor microbiota in patients with recurrent *Clostridioides difficile* infection that received FMT from healthy donors [28, 35], indicating that FMT has the potential to restore microbiota deficiencies in C-section infants even later in life.

In conclusion, limitations in our mechanistic understanding of the microbiome initialization and development process and its resilience to perturbation currently prevent a comprehensive assessment of the acute and lasting functional consequences of C-section for the microbiome and the host, as well as ultimately the health effects of the proposed microbiota restoration therapies.
